# Raptor breeding sites indicate high plant biodiversity in urban ecosystems

**DOI:** 10.1038/s41598-021-00556-4

**Published:** 2021-10-27

**Authors:** Haruki Natsukawa, Hiroki Yuasa, Shizuko Komuro, Fabrizio Sergio

**Affiliations:** 1 Graduate School of Environment and Information Sciences, Yokohama National University, 79-1 Tokiwadai, Hodogaya, Yokohama, Kanagawa Japan; 2 Graduate School of Media and Governance, Keio University, 5322 Endo, Fujisawa, Kanagawa Japan; 3 Independent Researcher, Tokyo, Japan; 4 Department of Conservation Biology, Estación Biológica de Doñana − CSIC, C/Americo Vespucio 26, 41092 Seville, Spain

**Keywords:** Biodiversity, Conservation biology

## Abstract

Preserving biodiversity in urban ecosystems has become an urgent conservation priority, given the rapid upsurge in global urbanization. As woody plants play essential ecological roles and provide psychological benefits to human city dwellers, their preservation is of particular interest to conservation scientists. However, considering that extensive censuses of woody plants are resource-intensive, a key accomplishment is to find reliable conservation proxies that can be quickly used to locate biologically diverse areas. Here, we test the idea that sites occupied by apex predators can indicate high overall biodiversity, including high diversity of woody plants. To this end, we surveyed woody plant species within 500 m of Northern Goshawk (*Accipiter gentilis*) breeding sites in urban ecosystems of Japan and compared them with non-breeding control sites without goshawks. We found that goshawks successfully identified and signposted high levels of richness, abundance, and diversity of woody plants. Our findings show that sites occupied by top predatory species could be exploited as conservation proxies for high plant diversity. Due to their exigent ecological requirements, we would expect apex predators to be tied to high biodiversity levels in many other urban ecosystems worldwide.

## Introduction

Globally, cities and towns are currently home to the majority of people. Approximately two-thirds of the global human population is predicted to be living in urbanized areas by 2025, and urban land cover is estimated to double between 2000 and 2025^1^. Thus, urbanization is widely recognized as a major threat to biodiversity because it leads to the loss and degradation of natural habitats. Accordingly, biodiversity is often lower in urban areas than in undeveloped natural areas^2,3^, although the former can offer occasional refuge to both common and threatened species^3,4^. Moreover, urban biodiversity can promote the health and well-being of city residents^5^, which emphasizes the well-recognized importance of biodiversity conservation in urban areas^6^. Thus, given the increasing concentration and growth of the human population in urban areas, maintaining the current biodiversity in urban ecosystems can be an effective way to improve the quality of life for many people^7^. Furthermore, high biodiversity values in urban ecosystems are a source of environmental education for urban residents. This process motivates citizens to maintain biodiversity, which ultimately contributes to the protection of other more natural ecosystems^3,6^. Therefore, the conservation of biodiversity in urban ecosystems is considered a key target for promoting biodiversity in general.

Biodiversity protection unavoidably entails the identification of biodiverse areas^8^. However, as biodiversity conservation is usually very resource-demanding, practical conservation action needs to be planned under limited time, budget, and human resource conditions^9^. These constraints have frequently imposed the necessity to use indicator species capable of flagging high levels of biodiversity in a cost-efficient manner^10^. Among potential indicator taxa, top predatory species, such as raptors, have been proposed to be efficient biodiversity surrogates e.g.^11,12^. As with other top predators, raptors have exigent, large-scale habitat requirements and are particularly sensitive to human-induced disturbances^13,14^. Thus, the preservation of raptor habitats could provide a protective umbrella for many other taxonomic groups. Furthermore, considering the charismatic demeanor of raptor species, they frequently serve as iconic species in global conservation programs^13,14^. Consequently, these species are subjected to an elevated level of field monitoring by both researchers and bird enthusiasts in various countries and regions^15^. This has led to the accumulation of valuable information on raptor distribution and, in principle, this information could be easily exploited to locate and protect sites with higher biodiversity. In addition, the charisma typical of these iconic species facilitates the approval and donation of funding for their conservation by citizens and governments^14^. Finally, raptor habitats are often protected by specific environmental laws that mandate the preservation of their habitats in different countries^16,17^. Therefore, if a raptor species is proven to act as an efficient biodiversity surrogate, it could be possible to exploit it to secure funding for conservation and to quickly identify sites of high conservation value and legally prioritize their preservation.

To date, only two studies have evaluated the usefulness of raptors as conservation surrogates for biodiversity in urban ecosystems. In one study, biodiversity was estimated by the richness of breeding birds^18^, employed as “background taxa” (the taxonomic group expected to benefit from the conservation of the surrogate species, following^10^), whereas the other study focused on wintering birds as background taxa^19^. In these assessments, there was a direct predator–prey relationship between the biodiversity surrogate (a bird-eating predator) and the background taxa (breeding or wintering birds). Ideally, the verification of the surrogacy function of a species should be conducted by relating sets of species that are not involved in direct ecological relations (e.g., predator–prey relations)^20^ (and references therein). This is because species involved in direct ecological relationships are more likely to be spatio-temporally related to each other (e.g., predators and prey fluctuating in parallel). In line with this idea, surrogacy performance is higher when the biodiversity surrogate and the background taxa belong to the same taxonomic group^21^. Thus, a stronger demonstration of the indicator capability of a potential biodiversity surrogate species requires testing of broader links between such species and background taxa that are as ecologically distant from it as possible (e.g., in this case, as trophically disconnected as possible and belonging to taxonomic groups that are as different as possible). Such demonstration would give us more confidence in the wider ecosystem-level value of the indicator species.

In this study, we tested the effectiveness of raptors as biodiversity surrogates in urban areas of Japan by using the Northern Goshawk (*Accipiter gentilis*, hereafter goshawk) as a potential surrogate and woody plants as a background taxonomic group. The goshawk is a medium-sized raptor that is distributed widely in the northern hemisphere^22^. The species preys primarily on birds and builds its nests in forest stands with low human disturbance^22^. In addition to its wide distributional range, the species meets the aforementioned advantages of charisma and iconicity^22^, which are important for exploiting it as a flagship species to leverage attention and funding for biodiversity conservation. Therefore, if this species proves to be suitable as a conservation surrogate, it could provide a basis for practical conservation in multiple countries and regions. In the present study, woody plants were used as indicators of overall biodiversity because they are tightly linked to the productivity of the entire ecosystem and the diversity of various taxonomic groups^23^. Further, they account for more than 90% of the global terrestrial biomass ^24^, and their diversity bears important consequences for the quality of life of human city dwellers^5^.

## Results

In the following analyses, biodiversity estimates were based on 17 489 woody plant individuals classified by species and site type (goshawk vs. control). The average woody plant species richness (number of species) in breeding and control sites was 35.3 ± 1.4 (mean ± SE) and 23.0 ± 4.2, respectively; the average abundances (number of individuals) were 402.0 ± 25.0 and 181.0 ± 29.6; and the average diversity values (Shannon–Wiener diversity index) were 3.7 ± 0.1 and 3.4 ± 0.1.

**Figure 1:**
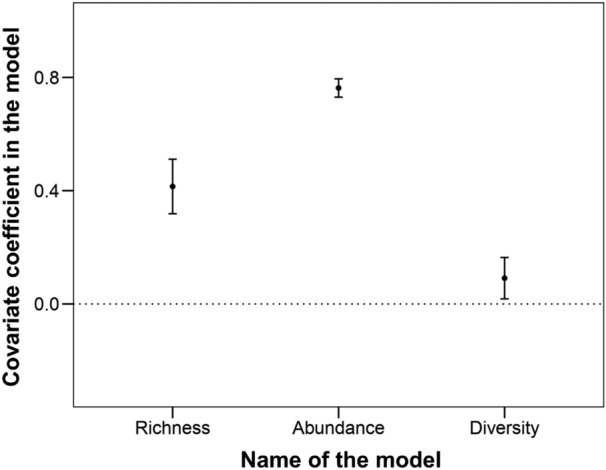
Estimated values of the regression coefficients and their 95% confidence intervals (CIs) for the generalized linear models used to examine the effect of goshawk presence/absence on the richness, abundance, and diversity of woody plants. When the 95% CIs excluded zero, the effects were regarded as statistically significant.

**Figure 2:**
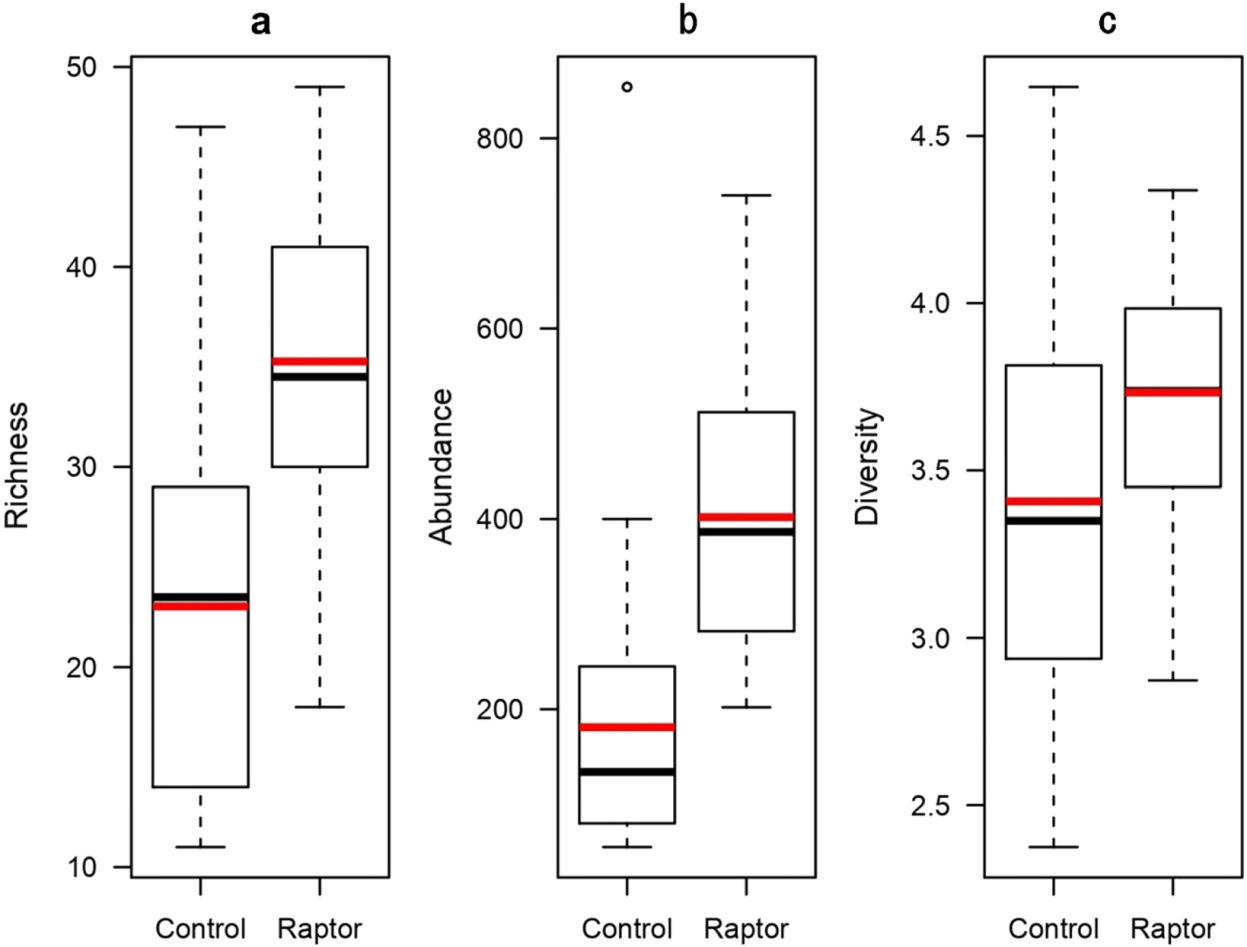
Box plots of descriptive statistics for woody plant species richness (panel **a**), abundance (panel **b**), and diversity (panel **c**) measured at goshawk breeding (raptor) and non-breeding (control) sites in urban areas of eastern Kanagawa, Japan. Box and whisker plots indicate the median with upper and lower quartiles. Red lines within boxes indicate mean values. Hollow circle indicates upper quartile (+ 1.5 times the interquartile range).

The generalized linear model (GLM) analyses to examine the effectiveness of goshawks as surrogates of woody plant diversity confirmed that woody plant richness, abundance, and diversity significantly increased with goshawk presence (Figs. 1 and 2). This implied that goshawk breeding sites can be a reliable proxy for woody plant biodiversity in these urban ecosystems. The results of these GLMs are summarized in Supplementary Table S1.

**Figure 3:**
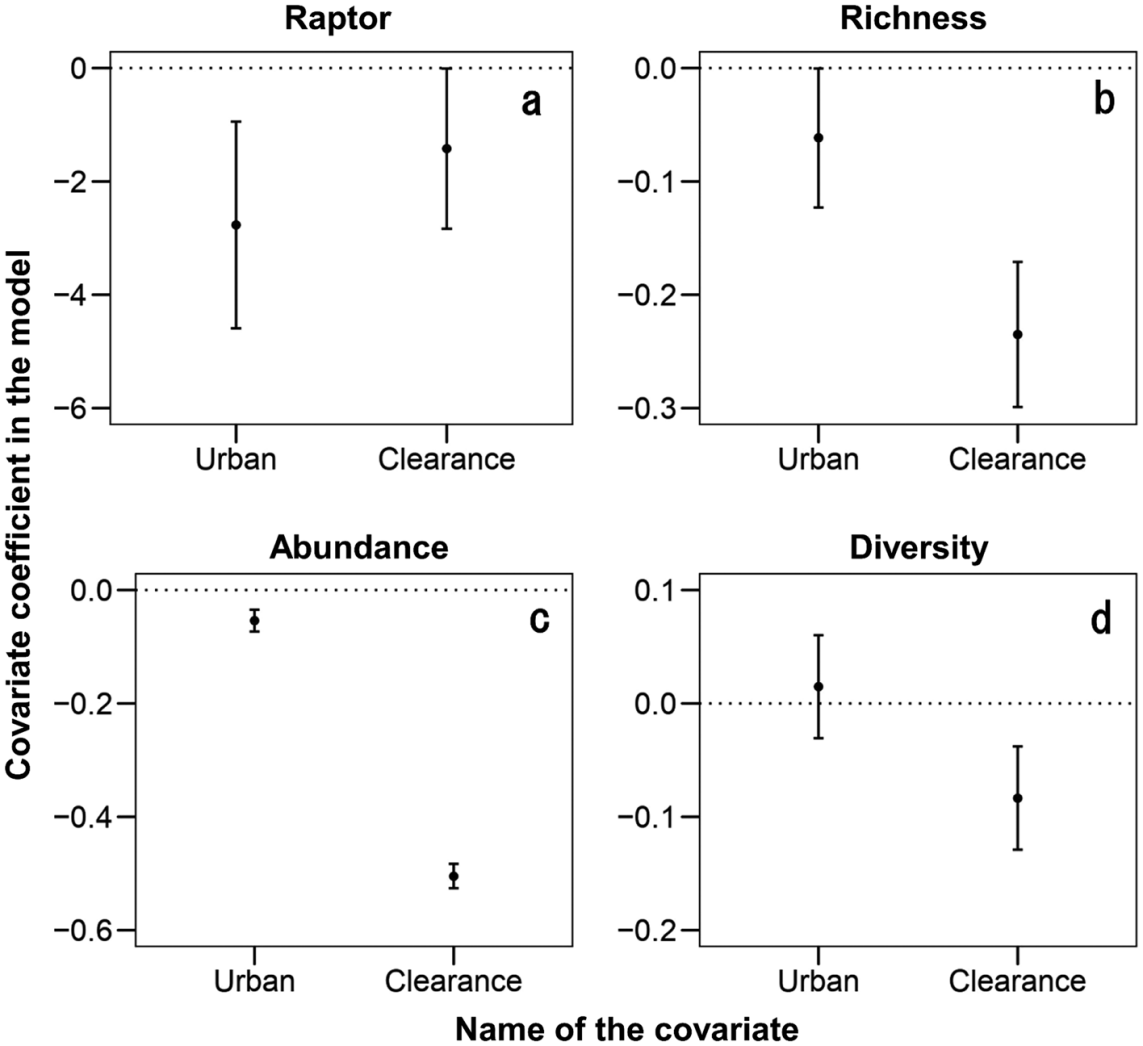
Estimated values of the regression coefficients and their 95% confidence intervals (CIs) from the generalized linear models used to identify the effect of environmental factors (percentage of urban areas and clearance of understory trees) on presence/absence of goshawk breeding sites (raptor, panel **a**) and on woody plant species richness (panel **b**), abundance (panel **c**), and diversity (panel **d**). When the 95% CIs excluded zero, the effects of covariates were regarded as statistically significant.

In the additional GLM analyses to identify the potential mechanisms behind surrogacy, the extent of urban areas and of forest clearance simultaneously affected goshawk occurrence and woody plant richness and abundance, whereas woody plant diversity was only related to forest clearance (Fig. 3). The summary of these GLMs is provided in Supplementary Table S2.

## Discussion

Our findings showed that goshawk breeding sites could be used as reliable conservation proxies for the richness, abundance, and diversity of woody plants in our studied urban ecosystems (Figs. 1, 2, and Supplementary Table S1). To our knowledge, this is the first empirical study demonstrating that the presence of a top predatory species is indicative of high diversity of trophically disconnected taxa in urban ecosystems. Consequently, the present study supports and extends the results of previous demonstrations of top predatory species as indicators of the diversity of trophically disconnected taxa, such as woody plants or fungi, in other far away regions and in more natural ecosystems^11,12^.

One possible interpretation of the surrogacy performance confirmed in the present study could be that the breeding site selection of goshawks incorporates features that also promote woody plant biodiversity in the urban ecosystems studied. Indeed, the main factors driving goshawk habitat selection (here, percentage of urban areas and level of clearance of understory trees) were basically the same as those promoting high plant richness, abundance, and diversity (Fig. 3 and Supplementary Table S2). Moreover, previous studies have obtained results compatible with these relationships: first, the diversity of woody plant species in urban ecosystems tends to increase in areas with lower urban land cover^25^; second, the clearance of understory trees is known to deteriorate plant biodiversity^26,27^. The matched response of goshawks and plant biodiversity to the same factors could be caused by the fact that the drivers of plant diversity simultaneously increase habitat suitability for goshawks. Goshawks typically nest in forests with low levels of disturbance (e.g., forests with no clear-cutting; reviewed in^28^), and management recommendations usually include the maintenance of understory trees in their nest stand^29^, which reduces the frequency of approach by human and mammalian predators. Given the small size of forest stands and the unavoidably high anthropogenic pressure in urban landscapes, the existence of less-disturbed and thus relatively well-preserved forest stands is especially important for goshawks in these areas. For example, goshawks in our study area typically select less-disturbed and larger forest patches and avoid intensively urbanized areas for breeding^30,31^. Overall, it appears that well-preserved forest fragments with high integrity of native plant biodiversity meet goshawks ecological requirements. Consequently, the goshawk preference for such habitat configurations could trigger the association between the presence of this species and higher biodiversity. Importantly, the results of the current study based on goshawks might be applicable to the breeding of other tree-nesting raptors in urban ecosystems, given that many other urban-breeding raptors have large home ranges and similar habitat selectivity (i.e., preference for less-disturbed habitat patches and the avoidance of intensive urbanization) (reviewed in^32^).

Understanding the mechanisms that underlie the indicator function of a species is important because it strengthens our confidence in their usage as surrogates^10^. The indicator function can be promoted by a spatial association between the surrogate species and biodiversity, as previously described. However, some predators can even act as causative agents of biodiversity or at least some components of biodiversity (reviewed in^14^). In the specific case of goshawks, a recent study showed that this species can impose a mix of positive and negative top-down effects on the distribution of other avian species, depending on whether they are potential goshawk prey or not, which would represent an indirect form of predator–prey interaction^33^. Thus, in this case, a mix of associative and causative processes could generate the frequent association of this species with some components of biodiversity^11,12,30,31^, which might justify its usage as a sentinel of the general health of forest ecosystems (e.g.^34^). However, it should be stressed that these processes might not apply in all ecosystems, even for the same species. For example, goshawks were not found to act as reliable biodiversity indicators in an agricultural ecosystem^35^. This suggests the need to test the indicator function of a species before its application to other ecosystems, rather than blindly accepting its surrogate potential based on studies conducted in different ecosystem types.

Overall, the results obtained have clear implications for applied conservation and management. Because of their appealing look, iconic charisma, frequently delicate conservation status, and sensitivity to human-induced disturbances, raptor species (including goshawks) are frequently monitored by professional and amateur ornithologists in many countries and regions^15^. In many cases, their habitats are already protected by environmental laws, ordinances, and guidelines^16,17^. This is particularly true for the preservation of goshawk breeding sites throughout their distribution range (reviewed in^22^). For example, the Japanese Ministry of the Environment has issued guidelines for the protection of goshawk breeding sites, and prefectures (including the Kanagawa prefecture of this study) even have their own locally fine-tuned protection guidelines. Therefore, because an abundance of information on the breeding sites and habitat characteristics of raptor species is available, this could represent an efficient tool to quickly identify biologically diverse sites and intensify their legal protection. This advantage is further emphasized by the fact that the predictive performance of goshawk occurrence better predicted three diversity measures of woody plants than land cover, which can be easily measured but is not associated with these social benefits (Supplementary Table S3). Furthermore, landowners are sometimes in favor of protecting raptors and their habitats on their lands, sometimes even without subsidies^36^. These sociological advantages could provide important benefits, when considering that many sites of conservation importance are private properties^37^, that many biodiversity-rich habitats are frequently not legally protected, especially in urban areas^38,39^, and that budgets for biodiversity conservation are often inadequate^40^. Finally, considering that many people find both predators and green spaces esthetically and psychologically appealing in urban environments^5,41,42^, a conservation strategy based on a raptor-indicator of tree diversity could provide a promising solution that exploits the flagship and umbrella concepts to attain a wider biodiversity target.

In conclusion, apex predators may or may not always be reliable biodiversity indicators, but well-chosen predators for which their indicator role is well substantiated could represent important tools for leveraging and fine-tuning conservation action. This scenario is based on the “talent-scout” capability of conservation practitioners capable of designing effective indicators, testing their reliability, and enacting their usage.

## Methods

### Surveyed areas

**Figure 4:**
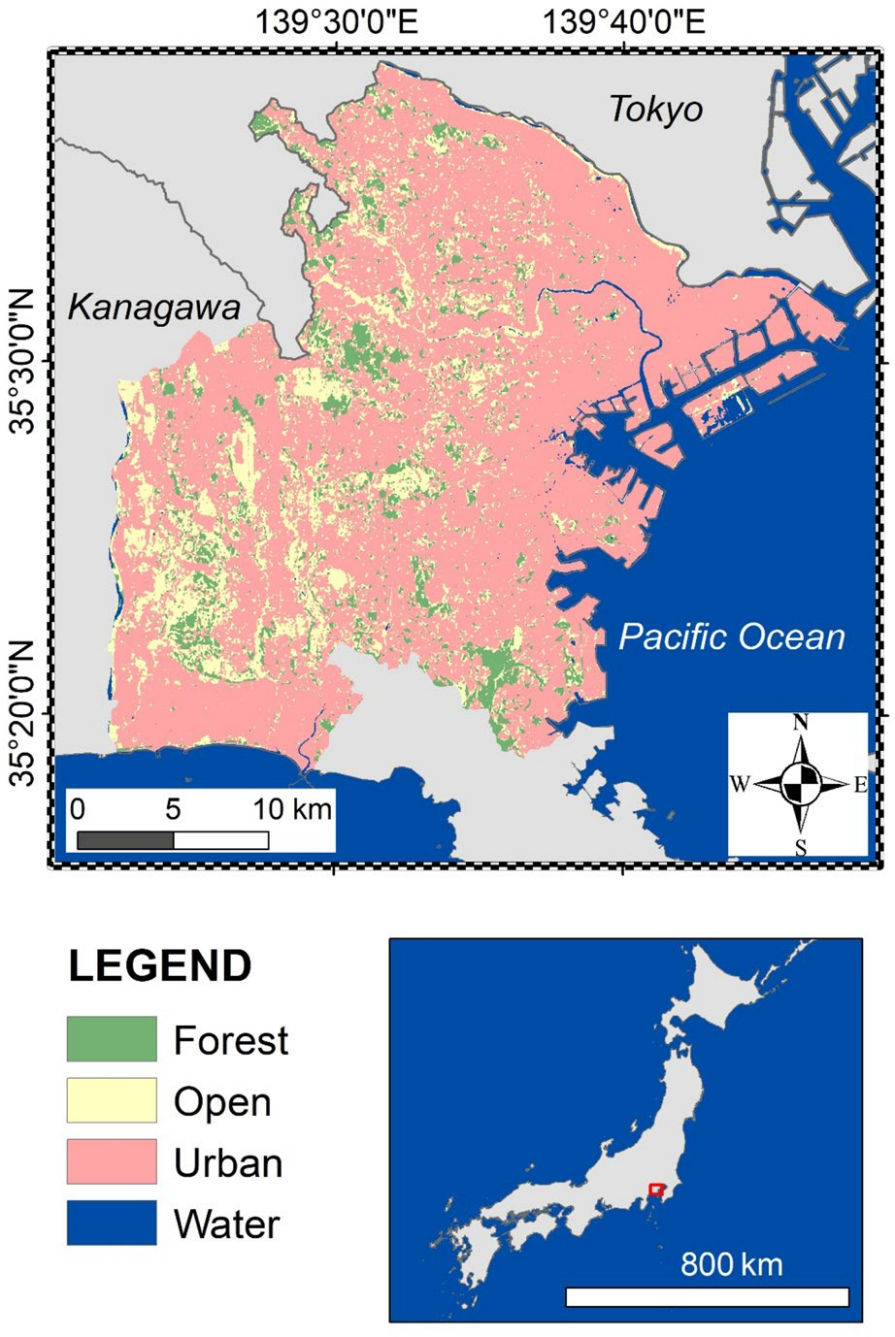
Study area for goshawk surveys in eastern Kanagawa, Japan (793 km^2^). This map was generated with ArcGIS 10.8, using the land cover map issued by the Japan Aerospace Exploration Agency (https://www.eorc.jaxa.jp/ALOS/lulc/jlulc_jpn.htm).

This study was conducted within an area of 793 km^2^ in eastern parts of Kanagawa, central Japan, which includes the cities of Kawasaki, Yokohama, Yamato, Zama, Ebina, Ayase, Fujisawa, and Chigasaki and the town of Samukawa (Fig. 4). The study area is located within the Tokyo-Yokohama district, which has the largest human population of all urban areas in the world and the third highest percentage of urban land use globally^43^. In terms of landscape composition, the surveyed area includes 13.0% forest, 11.8% open land (fields, paddy fields, and grasslands), 1.8% water (rivers and ponds), and 72.7% urban areas (buildings and paved roads). The topography is dominated by flatlands and gentle hills, with a maximum elevation of 159.4 m above sea level.

### Woody plant survey

We randomly selected 30 of the 37 goshawk breeding sites detected by^31^. For breeding sites that contained multiple alternative nests of the same pair, we only considered the latest-used nest. For each goshawk site or control location (see below), we performed the following: (1) we generated three random points in the forested areas within 500 m of the target location, and (2) surveyed all woody plant species and individuals (with a height ≥ 1.2 m) within 12 m of each of the three randomly selected points^44^. As we did not collect plant species to identify, special permission or guidelines to be followed were not required in our fieldwork. To avoid differences in richness and abundance owing to differences in the size of the forested area, the three selected points were plotted to be entirely covered by forests. In addition, to avoid spatially redundant sampling, the distance among these three points was constrained to be ≥ 50 m. Similar surveys were conducted at 30 randomly selected non-breeding sites, which were used as controls. The randomly selected non-breeding sites had a percentage of forested area and open land (total percentage of fields, paddies, and grasslands) that exceeded the lowest values at the goshawk breeding sites. These control non-breeding sites were thoroughly surveyed using the procedures described by^31^. The absence of goshawk breeding sites within a 1 km radius from these control sites was confirmed during the 2014–2020 period. To avoid spatial dependence, the distance between the breeding and control sites and among control sites was constrained to be ≥ 1 km.

### Statistical analysis

#### Quantification of woody plant diversity

The overall woody plant biodiversity was estimated by its species richness (number of species), total abundance (number of individuals), and diversity (Shannon–Wiener taxonomic diversity index^45^; hereafter, diversity). Liana plant species were excluded from these measurements. Furthermore, alien and ornamental species were also excluded from analysis, as conservation science usually targets the preservation of native species^46^. These values were calculated in R 4.0.3^47^ using the vegan package^48^.

#### Goshawk breeding sites as surrogates for woody plant diversity

To examine the efficacy of goshawk breeding sites as surrogates of woody plant biodiversity, we built three generalized linear models (GLMs^49^) with species richness, total abundance, and diversity as response variables and goshawk occurrence (goshawk breeding site vs. control) as a covariate. The error distribution and link function of the GLMs were Poisson and log link, respectively, for the models on richness and abundance and gamma and log link for the model on diversity.

#### Potential mechanisms underlying surrogacy

To investigate some of the potential mechanisms that could produce an association between goshawk presence and plant biodiversity, we used four additional GLMs to test the effect of two environmental factors (see below) on goshawk occurrence (comparison of goshawk vs. control sites) and on plant richness, abundance, and diversity. The model of goshawk occurrence was built with a Bernoulli distribution and logit link function (see the section Goshawk breeding sites as surrogates for woody plant diversity for richness, abundance, and diversity models). The rationale behind such analysis was to search for factors that simultaneously predicted goshawk occurrence and biodiversity levels, because identifying the mechanisms underlying surrogacy will increase our confidence in using biodiversity surrogates in conservation planning and action^10^. To this end, we fitted the percentage of urban areas within 500 m of each breeding/control site and the level of understory clearance for recreation, defined as the number of clear-cut plots included in the three plots 12 m in radius sampled for each breeding and non-breeding site, as covariates. These variables were chosen because they are known to potentially affect both goshawk breeding-site selection and woody plant diversity^25–28,30^. Land cover factors were measured using ArcMap 10.8 (ESRI, United States) and the land cover map issued by the Japan Aerospace Exploration Agency. The resolution of this map is 10 m^2^ and portrays the landscape structure of 2006–2011.

#### Procedure to fit GLMs

Intercepts and regression coefficients were identified by maximum likelihood estimation^49^. The estimated coefficients were considered to have significant effects on the response variables when the 95% confidence interval did not include zero^50^. Throughout, there was no indication of collinearity among covariates, as tested by examining variance inflation factors (VIFs^49^). Generally, a VIF > 10 indicates severe collinearity^51^, whereas all variables of this study had a VIF ≤ 1.79. Prior to fitting all GLMs, we examined the potential spatial autocorrelation (SAC^52^) in goshawk occurrence, species richness, total abundance, and diversity via the Moran’s I statistic^53^. Moran’s I ranges from − 1 to + 1, and values closer to zero indicate spatial independence (i.e., no SAC). Whenever Moran’s I statistic was significant, a spatial autocovariate was fitted to the model as an additional covariate, following^54^. Using this method, spatial autocorrelation was low but significant for goshawk occurrence (I = 0.15, P < 0.05) and the three biodiversity components (richness: I = 0.19, P < 0.05; abundance: I = 0.14, P < 0.05; diversity: I = 0.18, P < 0.05). Accordingly, spatial autocovariates were added to all of the GLMs. All statistical analyses were conducted in R 4.0.3^47^ using the packages rms^55^ and spdep^56^.

## Supplementary Information

Supplementary Information.

## Data Availability

Data will be available on reasonable request from the authors.
