# Supplementary material for: Raptor breeding sites indicate high plant biodiversity in urban ecosystems
Source: Sci Rep. 2021 Oct 27;11:21139. doi: 10.1038/s41598-021-00556-4 (PMC8551175; doi:10.1038/s41598-021-00556-4)
Supplement: Supplementary file 1 — Supplementary Information. [file 41598_2021_556_MOESM1_ESM.docx]

| **Supplementary Information**  **Table S1**  Intercept, coefficient estimates, and lower (LCI) and upper (UCI) 95 % confidence intervals from generalized linear models testing the usefulness of goshawk breeding sites as biodiversity surrogates in urban areas of eastern Kanagawa, Japan. | | | |
| --- | --- | --- | --- |
| Model and variable (sample size) | Estimate | LCI | UCI |
| **A. Response variable: richness (60) ^a^** |  |  |  |
| Intercept | 2.835 | 2.603 | 3.065 |
| Presence/absence of goshawks ^b^ | 0.415 | 0.319 | 0.512 |
| Spatial autocovariate ^c^ | 0.000 | 0.000 | 0.000 |
| **B. Response variable: abundance (60) ^a^** |  |  |  |
| Intercept | 4.770 | 4.703 | 4.837 |
| Presence/absence of goshawks ^b^ | 0.763 | 0.730 | 0.795 |
| Spatial autocovariate ^c^ | 0.000 | 0.000 | 0.000 |
| **C. Response variable: diversity (60) ^d^** |  |  |  |
| Intercept | 1.100 | 0.905 | 1.297 |
| Presence/absence of goshawks ^b^ | 0.091 | 0.018 | 0.164 |
| Spatial autocovariate ^c^ | 0.000 | 0.000 | 0.000 |
| ^a^ Generalized linear model with Poisson error and log link function  ^b^ 1 = goshawk breeding site; 0 = randomly selected control site  ^c^ Spatial autocovariate based on the coordinate information of each site  ^d^ Generalized linear model with gamma error and log link function | | | |

| **Table S2**  Intercept, coefficient estimates, and lower (LCI) and upper (UCI) 95 % confidence intervals from the generalized linear models used to identify the effects of environmental factors on breeding site selection by goshawks and on woody plant species richness, abundance, and diversity in urban areas of eastern Kanagawa, Japan. | | | |
| --- | --- | --- | --- |
| Model and variable (sample size) | Estimate | LCI | UCI |
| **A. Response variable: goshawk (60) ^a^** |  |  |  |
| Intercept | 0.440 | −3.590 | 5.046 |
| % cover of urban land ^b^ | −2.768 | −5.081 | −1.291 |
| Clearance of understory trees ^c^ | −1.421 | −3.083 | −0.124 |
| Spatial autocovariate ^d^ | −0.001 | −0.014 | 0.009 |
| **B. Response variable: richness (60) ^e^** |  |  |  |
| Intercept | 2.948 | 2.719 | 3.175 |
| % cover of urban land ^b^ | −0.062 | −0.123 | −0.001 |
| Clearance of understory trees ^c^ | −0.235 | −0.299 | −0.171 |
| Spatial autocovariate ^d^ | 0.000 | 0.000 | 0.000 |
| **C. Response variable: abundance (60) ^e^** |  |  |  |
| Intercept | 5.001 | 4.936 | 5.066 |
| % cover of urban land ^b^ | −0.054 | −0.073 | −0.035 |
| Clearance of understory trees ^c^ | −0.505 | −0.526 | −0.483 |
| Spatial autocovariate ^d^ | 0.000 | 0.000 | 0.000 |
| **D. Response variable: diversity (60) ^f^** |  |  |  |
| Intercept | 1.085 | 0.903 | 1.268 |
| % cover of urban land ^b^ | 0.015 | −0.029 | 0.058 |
| Clearance of understory trees ^c^ | −0.084 | −0.129 | −0.038 |
| Spatial autocovariate ^d^ | 0.000 | 0.000 | 0.000 |
| ^a^ Generalized linear model with Bernoulli error and logit link function  ^b^ Percentage of urban areas within 500 m from each site  ^c^ Level of understory clearance within 500 m from each site  ^d^ Spatial autocovariate based on the coordinate information of each site  ^e^ Generalized linear model with Poisson error and log link function  ^f^ Generalized linear model with gamma error and log link function | | | |

| **Table S3**  Akaike Information Criterion (AIC) from the generalized linear models used to identify the effects of environmental factors on woody plant species richness, abundance, and diversity in urban areas of eastern Kanagawa, Japan. | |
| --- | --- |
| Model and variable (sample size) | AIC |
| **A. Response variable: richness (60) ^a^** |  |
| Presence/absence of breeding sites ^b^ | 480.199 ^e^ |
| % cover of urban land ^c^ | 481.547 ^f^ |
| **B. Response variable: abundance (60) ^a^** |  |
| Presence/absence of breeding sites ^b^ | 4632.181 ^e^ |
| % cover of urban land ^c^ | 5076.441 ^f^ |
| **C. Response variable: diversity (60) ^d^** |  |
| Presence/absence of breeding sites ^b^ | 96.212 ^e^ |
| % cover of urban land ^c^ | 98.413 ^f^ |
| ^a^ Generalized linear model with Poisson error and log link function  ^b^ 1 = goshawk breeding site; 0 = randomly selected control site  ^c^ Percentage of urban areas within 500 m from each site  ^d^ Generalized linear model with gamma error and log link function  ^e^ AIC for models estimated by presence/absence of breeding site and spatial autocovariate  ^f^ AIC for models estimated by percentage cover of urban areas and spatial autocovariate | |
